# The level of consciousness and mental reactions of children after acute brain injury (interdisciplinary rehabilitation)

**DOI:** 10.1192/j.eurpsy.2021.1343

**Published:** 2021-08-13

**Authors:** A. Kamelkova, D. Martyshevskaya, A. Zakrepina, Y. Sidneva

**Affiliations:** 1 Department Of Rehabilitation, Clinical and Research Institute of Emergency Pediatric Surgery and Trauma (CRIEPST), Moscow, Russian Federation; 2 Laboratory Of Psychological And Pedagogical Research And Technologies For Special Education Of Persons With Intellectual Disabilities, The Federal State Budget Scientific Institution “Institute of Special Education of the Russian Academy of Education”, Moscow, Russian Federation; 3 The Department Of Rehabilitation, Clinical and Research Institute of Emergency Pediatric Surgery and Trauma (CRIEPST), Moscow, Russian Federation; 4 Psychiatric Research Group, N.N.Burdenko National Medical Research Center of Neurosurgery, Moscow, Russian Federation

**Keywords:** interdisciplinary approach, child rehabilitation, mental recovery, minimal consciousness

## Abstract

**Introduction:**

The process of recovery of mental reactions in children after acute traumatic brain injury is determined by complex methods with an interdisciplinary approach. Studies of emotional, communicative and behavioral reactions are based on an assessment by a psychiatrist and a teacher-defectologist.

**Objectives:**

To study mental reactions and identify predictors of positive recovery of consciousness after acute brain injury in children in early rehabilitation.

**Methods:**

48 children (14–36 months) with acute severe traumatic brain injury who were admitted for treatment and rehabilitation (in CRIEPST). Methods: psychiatric and pedagogical examinations; also - neuroimaging data and others.

**Results:**

Three groups of children were identified, depending on the different severity of emotional, communicative and behavioral indicators: Group 1 (11%): The level of consciousness is minimal positive. Reactions: stable gaze fixation; emotional reaction to sound (smile) and the face of an adult; short-term tracking of the gaze of the object; the ability to touch an object and hold it; sits himself. Group 2 (33%): The level of consciousness is minimal positive / negative, with an advantage of positive. Reactions: unstable gaze fixation; emotional reaction and involuntary movements to sound; reflex seizure of an object; sits with support. Group 3 (56%): The level of consciousness is minimal negative. Reactions: no emotional reactions, low motor and sensorimotor activity.

**Conclusions:**

Predictors of emotional-communicative and behavioral indicators of recovery of the level of consciousness were identified: sensory and motor, cognitive and socially-oriented. These predictors are the basis for choosing a rehabilitation program with interdisciplinary support and a treatment strategy.

